# The 3M^TM^ Cavilon^TM^ barrier prevents erasure of surgical skin markings with removal of povidone iodine adhesive draping

**DOI:** 10.1002/ski2.31

**Published:** 2021-05-02

**Authors:** T. Oshima, A. Sakamoto, T. Noguchi, S. Matsuda

**Affiliations:** ^1^ Department of Orthopaedic Surgery Noe Hospital Osaka Japan; ^2^ Department of Orthopaedic Surgery Graduate School of Medicine, Kyoto University Kyoto Japan

## Abstract

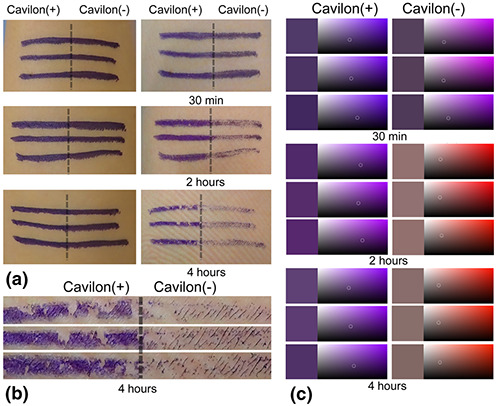

The 3M Cavilon barrier is a no‐sting film which acts as a physical barrier to protect the skin from friction and contamination.The 3M Cavilon barrier prevents erasure of surgical skin markings with removal of povidone iodine adhesive draping.

1

Dear Editor,

Surgical skin markers usually resemble a felt‐tipped pen, with ink flowing from an internal reservoir as the tip of the marker is brushed against the skin surface. Skin marking is used for several purposes, including marking the correct site before surgery, planning the surgical approach, and ensuring correct anatomical skin suturing after surgery.^1^, ^2^ Adhesive drapes are used during surgery to lower the rate of infection.^3^ When povidone iodine adhesive drapes are removed to allow suturing of the operative wound, markings are often removed along with the drape. The 3M^TM^ Cavilon^TM^ barrier (3M Health Care) is a no‐sting film which acts as a physical barrier to protect the skin from friction and contamination.^4^ We noticed that use of this barrier prevents erasure of skin markings, and we designed an experiment to attempt to clarify this beneficial effect. One Asian volunteer (first author, T.O.) completed the experiment. The institutional review board of Noe Hospital approved this minimally invasive experiment without deliberation.

The anterior aspect of the volunteer's forearm^1^ was inspected and noted to be moist and not hairy. The anterior forearm was washed with soap, then rinsed with tap water and dried. The site was disinfected three times using povidone iodine (10% vol/vol) and allowed to air dry. A commercially available surgical marker (Nesco Dermark®; Alfresa Phama Corporation) was used to mark nine transverse lines on the volunteer's forearm. The lines were about 4 cm in length. After the ink was dried, the 3M^TM^ Cavilon^TM^ barrier (applicator stick) was applied over the left half of each line. The drape was removed from three lines at 30 min, another three lines at 2 h, and the final three lines at 4 h. The lines were inspected visually and photographed using a digital camera. The colour of the photographic images was averaged, given a colour hex code and plotted.

At 30 min, there was no obvious visual difference between the lines that did and did not have the 3M^TM^ Cavilon^TM^ barrier applied (Figure 1a). At 2 h, the colour of the line was slightly faded with the 3M^TM^ Cavilon^TM^ barrier. At 4 h, there was an obvious visual difference, with the 3M^TM^ Cavilon^TM^ barrier preserving the surgical skin markings (Figure 1a, b). The average colour of the lines after removal of the drape at 30 min was purple (similar to the colour at application) with and without use of the 3M^TM^ Cavilon^TM^ barrier. The average colour with the barrier after removal of the drape at 2 and 4 h was purple. The plotted average colour without the barrier at 2 and 4 h was pale red to black (Figure 1c).

**FIGURE 1:**
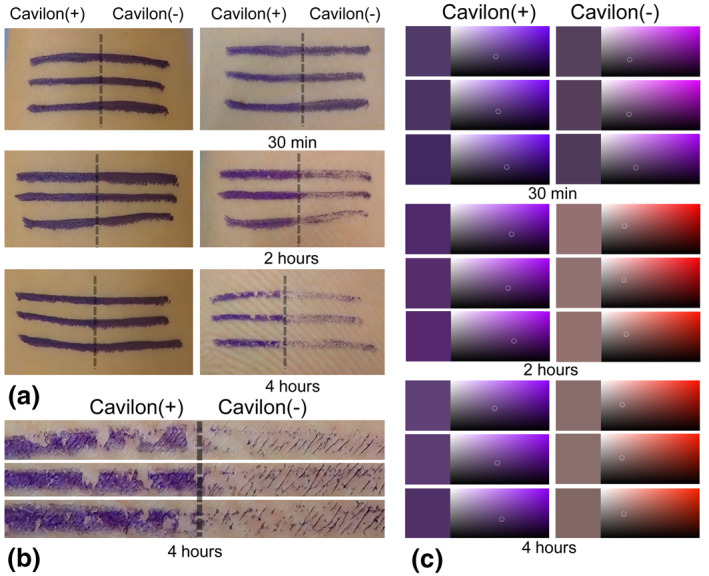
Surgical skin markings on the volunteer's forearm. (a) Markings before adhesive iodine draping are shown in the left‐hand panels. The 3M^TM^ Cavilon^TM^ barrier is applied to the left side of each line. The right‐hand panels show the lines after removal of the adhesive povidone iodine drape at 30 min (top), 2 h (middle), and 4 h (bottom).(b) Magnified view of the portion of the forearm with drape removal at 4 h.(c) Each of the lines in the right‐hand panels of (a) is given a hex colour code; these codes are then plotted (small white circles)

Our results support the use of the 3M^TM^ Cavilon^TM^ barrier to protect surgical markings. The degree of prevention varies according to skin conditions, such as temperature and the presence of sweat. Skin conditions may also differ in patients undergoing general anaesthesia versus local analgesia. In addition, there are many types of surgical skin markers, and the type could certainly affect the deterioration or preservation of markings.

Different types of cleansers may also affect skin markings differently. In addition to povidone iodine, chlorhexidine is widely used as a disinfectant for surgery. Some types of surgical skin markings fade after cleansing with chlorhexidine,^5^ so repeating the same experiment with chlorhexidine instead of povidone iodine would provide useful information for comparison. It would be interesting to see whether the 3M^TM^ Cavilon^TM^ barrier, applied before or after marking the skin, affects erasure of skin markings when chlorhexidine is used for cleansing, even without an adhesive povidone iodine drape.

In summary, the 3M^TM^ Cavilon^TM^ barrier prevents erasure of surgical skin markings when povidone iodine adhesive draping is removed. The efficacy of this prevention may differ according to skin condition, the type of surgical skin marker used, or the type of cleanser used. Our experiment confirmed our clinical experience that the 3M^TM^ Cavilon^TM^ barrier prevents loss of markings. The barrier is easy to use and effective at preventing erasure of surgical skin markings when povidone iodine adhesive drapings are used for surgery.

## CONFLICT OF INTERESTS

The authors declare that there are no conflict of interests.
